# An Unusual Etiology for Elevation of Activated Partial Thromboplastin Time (aPTT) in SLE: Acquired Hemophilia and Lupus Anticoagulant

**DOI:** 10.1155/2013/521785

**Published:** 2013-09-26

**Authors:** Srikanth Seethala, Nathaniel P. Collins, George Comerci

**Affiliations:** Department of Internal Medicine, University of New Mexico, Albuquerque, NM 87131, USA

## Abstract

A 60-year-old female who has a history significant for diabetes, depression, and rheumatoid arthritis presented with a progressively enlarging hematoma of the left upper extremity. She was found to have an enlarging hematoma and an isolated elevation of activated partial thromboplastin time (aPTT). Lab work-up revealed low factor VIII activity levels and inhibitor titers at 13.38 Bethesda units (BU). Dilute Russell's viper venom time (dRVVT) revealed a lupus anticoagulant. Hemostasis was achieved with factor VIII inhibitor bypassing activity (FEIBA) and inhibitor eradication with-rituxan after the failure of first-line treatment with cyclophosphamide and prednisone.

## 1. Introduction

Acquired hemophilia is a rare cause of aPTT elevation. It is associated with rheumatological conditions like systemic lupus erythematosus (SLE). Lupus anticoagulant (LA) may also increase the aPTT and be associated with many of the same rheumatological conditions. Both factor VIII inhibitors and LA can coexist in the same patient leading to a challenge in determining the etiology of an elevated aPTT.

## 2. Case

A 60-year-old female with a medical history significant for seronegative rheumatoid arthritis (RA), depression, diabetes, and hypothyroidism presented to the emergency department (ED) for abdominal pain. A thorough evaluation failed to reveal any significant abnormalities. Her hemoglobin (Hb) was 11.8 gm/dL, hematocrit (hct) was 36%, and platelet count is 445 K/*μ*L. Her ED course was complicated by multiple phlebotomies because of difficult venous access. She was subsequently discharged from the ED but three days later returned for progressive swelling, pain, numbness, and tingling in the left forearm, aggravated by wrist flexion and with minimal relief at rest. She denied any symptoms of infection, arm trauma, vigorous exercise of the upper extremity, or intravenous drug abuse. She noted that her symptoms began after multiple phlebotomy attempts in the ED. Her home medications included cyclobenzaprine, levothyroxine, trazodone, multivitamins, and nonsteroidal anti-inflammatory drugs (NSAIDS) as needed for RA. She denied the use of cytotoxic therapy, clopidogrel, aspirin or any other over the counter medications for the RA. Her physical exam revealed multiple ecchymosis of the left forearm extending from the elbow joint to wrist. She also was extremely tender upon deep palpation of the forearm. Her repeat lab work-up showed an Hct of 36, white blood cell count (WBC) of 12.6 × 10^3^cells/*μ*L, platelet count of 279 K/*μ*L, and normal blood chemistry. Her prothrombin time (PT) was 12.6 seconds, and her international normalized ratio (INR) was 0.98; activated partial thromboplastin time (aPTT) was not measured during this episode. Roentgenography (X-ray) of the left forearm revealed the possibility of a compartment syndrome, and the patient was taken to surgery. Intraoperatively, a contused brachioradialis muscle was found without evidence of a significant hematoma or an arterial tear. A fasciotomy was performed with hemostasis. Several hours postoperatively, the dressing was blood-soaked and required two changes overnight. By the following postoperative day, her Hb had dropped from 12 to 8.3 gm/dL, and her Hct had dropped from 36% to 25%. The patient received fresh frozen plasma (FFP) but this failed to control the bleeding. Platelet function studies showed no significant abnormalities. Her coagulation panel revealed a PT of 13.8 sec, an INR of 1.06, an aPTT of 63 sec, a fibrinogen level of 317 mg/dL (170–470), and a d-dimer of 1.73 *μ*g/L (<0.67 *μ*g/mL). Given the isolated aPTT elevation along with the history of rheumatoid arthritis, acquired hemophilia was suspected. Factor VIII activity levels and von Willebrand activity levels were sent. Factor VIII activity levels came back less than 0.1, and inhibitor titers came back at 13.38 BU. Chromogenic factor VIII levels were not performed. Hematology service was consulted. Hemostasis was achieved with activated prothrombin complex concentrates (aPCC or FEIBA) at a dose of 100 units/kg twice daily. Prednisone was started at a dose of 1 mg/kg/day for inhibitor eradication. The hexagonal phospholipid neutralization assay was positive for the lupus anticoagulant (LA) that was confirmed by dilute Russell's viper venom time (dRVVT).

The patient's treatment response (prednisone) was monitored by following factor VIII activity levels and inhibitor titers. Subsequent consultation and evaluation by rheumatologist led to a diagnosis of systemic lupus erythematosus (SLE), and hydroxychloroquine was initiated. After one week on prednisone, the inhibitor titer increased from 13.38 to 21.76. Therefore, therapy with cyclophosphamide 2 mg/kg/day was initiated. Unfortunately, one week after starting cyclophosphamide, the inhibitor titers increased to 34.25 BU. Given her failure to respond to first-line treatment, rituximab was initiated at a 375 mg/m^2^ dose weekly for four weeks. After the first dose, inhibitor titers dropped to 21.8 BU, along with a decrease in aPTT. This drop in aPTT suggested that the initial aPTT value was secondary only to acquired hemophilia A (AHA). FEIBA which had been given once a day was discontinued after the drop in aPTT. The patient was discharged and followed with aPTT and inhibitor titers. Her postdischarge course was complicated by an episode of febrile neutropenia that was successfully treated.

## 3. Discussion

Acquired hemophilia A or acquired factor VIII inhibitor (AHA) is a rare autoimmune disorder that is secondary to development of autoantibodies to factor VIII. Prevalence of AHA is reported in one to four cases per million per year. It has a bimodal age distribution with the first peak in the second to third decades of life. This peak is primarily due to postpartum acquired hemophilia, and the second major peak is between 65 and 85 years [1].

Factor VIII has a domain structure of A_1_-a_1_-A_2_-B-a_3_-A_3_-C_1_-C_2_. In acquired hemophilia (AH), IgG1 and IgG4 autoantibodies are developed against epitopes within the domains of A_2_, A_3_, and C_2_. The mechanism of action of antifactor (AF) VIII antibodies depends on target epitope location. Anti-A_3_ antibodies will prevent interaction of factor VIII with factor IXa. Anti-C_2_ antibody inhibits the binding of factor VIII to phospholipids and von Willebrand factor (VWF). Finally, anti-A_2_ & A_3_ antibodies interfere with factor VIII binding to factor X and factor IXa [2]. A correlation has not been established between the severity of the disease and the specific epitope involved in the disease process.

The clinical presentation of AHA is quite variable presenting as an incidental finding or as dramatic as a life-threatening hemorrhage. The most common presentations include easy bruisability, muscular hematomas, hematuria, intracerebral hemorrhage, gastrointestinal bleeding, epistaxis, or postpartum hemorrhage. In contrast to congenital hemophilia, hemarthrosis is not a part of the clinical presentation [1, 3, 4]. 

A high index of clinical suspicion is needed to diagnose AHA. It is the most common of all acquired hemophilia, and an isolated elevation of aPTT in a bleeding patient should raise the suspicion of AHA. Once the diagnosis is suspected, mixing studies should be performed. The aPTT will not be corrected after mixing the patient's plasma with the plasma that contains 100% of clotting factors (1 : 1 ratio) in the presence of an inhibitor, while in factor deficiency it will normalize. Chromogenic factor VIII assays may also be performed once the diagnosis is suspected as these assays are not affected by inhibitors. In addition, acquired factor VIII antibodies follow second-order kinetics. Precipitation is both time and temperature dependent. It is very crucial to perform a timed inhibitor screen particularly in cases of low inhibitor titers [1, 5–7]. It is also crucial to exclude other causes of aPTT elevation like heparin and lupus anticoagulant. Heparin contamination is not likely if the reptilase time is normal and can be confirmed by correction of aPTT after addition of heparinase or protamine. Anti-Xa activity will also help to exclude heparin contamination [6].

Lupus anticoagulant (LA) also causes isolated elevation of aPTT; moreover, it also shares common associated conditions like SLE. As in this case, AHA and lupus anticoagulant can coexist in the same patient. LA can also decrease the plasma factor VIII levels and can make the diagnosis more challenging. Sometimes differentiation between these two conditions can be difficult as both conditions can cause an isolated aPTT elevation that will not correct on mixing studies. The clinical presentation is very helpful to differentiate between these two conditions. A patient presenting with a clinical picture of thrombosis will make LA more likely, while a presentation with bleeding should raise suspicion for AHA. During an isolated aPTT elevation without any clinical findings, the diagnosis can be more challenging. In these scenarios, serial dilutions of the patient's plasma might provide some insight. On serial dilutions, the clotting time will shorten and follow a nonlinear pattern when LA is present, while clotting time will follow a linear pattern that is parallel to the control if factor VIII inhibitor is present [5]. Adding phospholipids will decrease aPTT with LA but not with factor VIII inhibitors. Factor VIII activity levels may be low with LA, but a very low or undetectable level makes AHA more likely. Finally, ELISA tests have been shown to accurately detect and determine inhibitors [4, 5]. 

The management of AHA has two steps. The first step is to achieve hemostasis. The treatment of choice depends on the clinical condition. In severe and life-threatening conditions, factor VIII bypassing agents should be used. These are activated prothrombin complex concentrates (aPCC or FEIBA) and recombinant factor VIIa (rVIIa). FEIBA dose ranges from 50 to 100 IU/Kg every 8–12 hours with caution for doses greater than 200 IU/KG/day as disseminated intravascular coagulation (DIC) may occur. An anamnestic response with increase in factor VIII titer has been reported. Also, antifibrinolytic agents should be avoided for 12 hours. Factor VIIa hemostasis can be achieved with a dose of 90 *μ*g/kg intravenously every 2-3 hours or a continuous infusion at 8–50 mg/kg/h. Caution must be taken when administering rVIIa in patients with atherosclerotic disease, sepsis, and DIC. Both of these agents have equal efficacy, and the choice of agent to use is based on availability and physician's experience. In minor bleeding episodes such as mucosal hemorrhagesand with inhibitor titers <5 BU, desmopressin, and factor VIII concentrates can be used [1, 4, 7]. 

The second step is inhibitor eradication. All patients with AHA should be treated with steroids alone (1 mg/kg/day for 4–6 weeks) or steroids with cyclophosphamide (1-2 mg/kg/day for a maximum of 6 weeks). A benefit and risk assessment should be done before initiation of cytotoxic therapy. If the inhibitor titers remain significantly elevated after 3–6 weeks of first-line agents, alternative therapy with rituximab, immunoadsorption, other cytotoxic agents (azathioprine, vincristine, and calcineurin inhibitors), or combination therapy should be considered [4, 7].

A complete response is defined as an undetectable inhibitor level and a normal factor VIII activity level, while a sustained response is defined as an undetectable inhibitor level and a factor VIII activity level greater than 50% after inhibitor eradication treatment. It is recommended to follow these patients with haemogram, aPTT, inhibitor titer, and factor VIII activity at least twice weekly while hospitalized and then once weekly as out-patients for 6 weeks. Once a complete response has been achieved, labs can be assessed monthly for the first 6 months and then every 2-3 months for the next 6 months. Patients should be monitored every 6 months for signs of relapse [4].

Relapses can occur in 20% of the cases, the median time to relapse being about 7–9 months. A second complete remission can be achieved in at least half of the patients. In some patients, immunosuppressive therapy should be continued for life in order to prevent relapse [8].
